# Is Supplementation with Micronutrients Still Necessary during Pregnancy? A Review

**DOI:** 10.3390/nu13093134

**Published:** 2021-09-08

**Authors:** Sonia Santander Ballestín, Marta Isabel Giménez Campos, Jara Ballestín Ballestín, María José Luesma Bartolomé

**Affiliations:** 1 Department of Pharmacology, Physiology and Legal and Forensic Medicine, Faculty of Medicine, University of Zaragoza, 50009 Zaragoza, Spain; 2 Department of Gynecology and Obstetrics, Hospital San Pedro, 26006 Logroño, Spain; martagimc@gmail.com; 3 Bioprocess Innovation Unit, ViraTherapeutics GmbH, 6063 Rum, Austria; jaraballestin@gmail.com; 4 Department of Human Anatomy and Histology, Faculty of Science, University of Zaragoza, 50009 Zaragoza, Spain; mjluesma@unizar.es

**Keywords:** pregnancy, vitamins, minerals, supplementation, requirement, maternal and fetal health

## Abstract

Introduction: Proper nutrition during pregnancy is important to prevent nutritional imbalances that interfere with pregnancy. Micronutrients play critical roles in embryogenesis, fetal growth, and maternal health, as energy, protein, vitamin, and mineral needs can increase during pregnancy. Increased needs can be met by increasing the intake of dietary micronutrients. Severe micronutrient deficiency or excess during pregnancy can have negative effects on fetal growth (intrauterine growth retardation, low birth weight, or congenital malformations) and pregnancy development (pre-eclampsia or gestational diabetes). We investigate whether it is necessary to continue micronutrient supplementation during pregnancy to improve women’s health in this stage and whether this supplementation could prevent and control pathologies associated with pregnancy. Aim: The present review aims to summarize evidence on the effects of nutritional deficiencies on maternal and newborn morbidity. Methods: This aim is addressed by critically reviewing results from published studies on supplementation with different nutrients during pregnancy. For this, major scientific databases, scientific texts, and official webpages have been consulted. PubMed searches using the terms “pregnancy” OR “maternal-fetal health” AND “vitamins” OR “minerals” OR “supplementation” AND “requirement” OR “deficiency nutrients” were performed. Results: There are accepted interventions during pregnancy, such as folic acid supplementation to prevent congenital neural tube defects, potassium iodide supplementation to correct neurodevelopment, and oral iron supplementation during the second half of pregnancy to reduce the risk of maternal anemia and iron deficiency. A number of micronutrients have also been associated with pre-eclampsia, gestational diabetes mellitus, and nausea and vomiting in pregnancy. In general, experimental studies are necessary to demonstrate the benefits of supplementation with different micronutrients and to adjust the recommended daily doses and the recommended periconceptional nutrition for mothers. Conclusions: Presently, there is evidence of the benefits of micronutrient supplementation in perinatal results, but indiscriminate use is discouraged due to the fact that the side effects of excessive doses are not known. Evidence supports the idea that micronutrient deficiencies negatively affect maternal health and the outcome of pregnancy. No single micronutrient is responsible for the adverse effects; thus, supplementing or correcting one deficiency will not be very effective while other deficiencies exist.

## 1. Introduction

Pregnancy represents a challenge from a nutritional perspective, given that micronutrient intake during the periods of periconception and pregnancy influences the mother’s health and the development of fetal organs [1,2]. It is known that, during pregnancy, a woman undergoes a series of physiological changes in her body, mainly at the level of her endocrine, digestive, cardiovascular, hematological, respiratory, and renal systems. This new situation entails an increase in the demands for energy, proteins, vitamins, and minerals. Balanced nutrition is important during the whole pregnancy and even in the periconceptional period, because the period before pregnancy is critically important for the health of a woman and her infant [3]. Therefore, the requirements will be higher compared to those of a healthy nonpregnant woman [4], and adequate maternal dietary intake is essential. Thus, during this stage, it is relevant to monitor the mother’s diet and ensure adequate nutritional intake to ensure sufficient metabolism and appropriate fetal development [5,6].

It is known that the fetus completely depends on the mother for its growth and development; therefore, the general physical condition of the mother will directly affect its health and chances of survival [7,8,9,10,11,12]. Recent research proposes that deficiencies or excesses of some nutrients are associated with problems in fetal growth and development, complications during pregnancy, and alterations in the later development of children. It is suggested that some adult diseases, as well as possible health alterations suffered by women who had deficiencies during pregnancy, have a fetal origin [7,13,14]. Deficiencies during pregnancy have also been related to the mother being in worse nutritional condition at the beginning of gestation, so there is great value to improving the prenatal diet and/or giving nutritional supplements [1,11].

According to the current recommendations, pregnant women should consume a healthy, balanced diet consistent with guidelines on healthy eating to guarantee the right amount of energy and nutrients, as well as an adequate supply of vitamins and minerals. In line with the majority of medical associations and WHO (World Health Organization) statements, routine usage of dietary supplements by all pregnant women is not recommended [15,16]. Thus, it is also stated in the guidelines and recommendations that a balanced diet should be a priority and, of course, if needed some supplementation should be provided.

Micronutrients are chemical substances that, when ingested in small quantities, allow us to regulate the metabolic and biochemical processes of the body [17]. Vitamins and minerals, referred to collectively as micronutrients, also including fatty acids, have important influences on the health of pregnant women and growing fetuses. Deficits in or lack of any of them can lead to growth deficiencies, problems in the development of cognitive and physiological functions, and immunodeficiencies.

**Figure 1:**
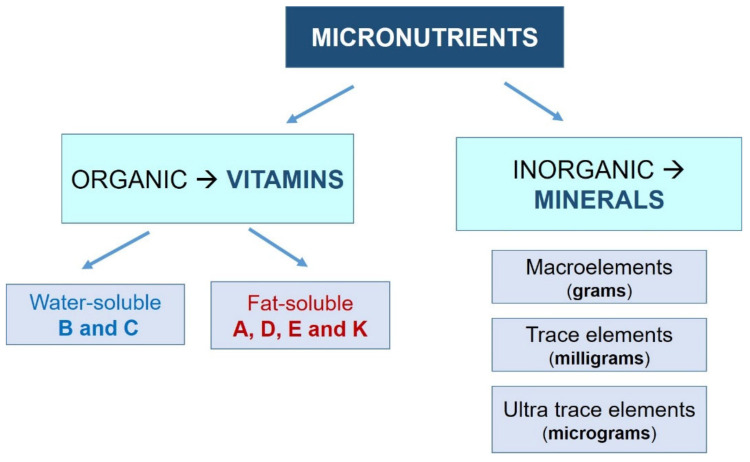
Classification system for the different micronutrients.

However, it is important to note that an excess of them will also negatively affect our health, which highlights the importance of establishing an appropriate dose for each specific situation [1]. Vitamins and minerals are defined as follows (Figure 1):

- Vitamins: Organic molecules necessary in small quantities for the proper functioning of the metabolism. Most vitamins are essential nutrients, so they must be ingested from external sources. Depending on their solubility, they are classified into two groups:
  - Water-soluble (they dissolve in water): vitamin B (B1, B2, B3, B6, and B12) and vitamin C.
  - Fat-soluble (they dissolve in fatty acids): vitamins A, D, E, and K.
- Minerals: Inorganic substances essential for growth and health. All of them are essential nutrients. They are usually classified into three groups, depending on their demand:
  - Macroelements: sodium, potassium, calcium, phosphorus, magnesium, chlorine, and sulfur. The human body demands these minerals in greater amounts (measured in grams).
  - Trace elements: iron, fluorine, iodine, manganese, cobalt, copper, and zinc. They are needed in lower amounts (measured in milligrams).
  - Ultratrace elements: silicon, nickel, chromium, lithium, molybdenum, and selenium. The human body requires these in very small amounts (on the order of micrograms).

These minerals are found in food in various forms, mixed or combined with different macronutrients. As an example of the necessity of minerals, note that a deficiency of β-carotene, magnesium, zinc, and/or calcium, among others, could increase the risk of suffering from pre-eclampsia. It remains to be studied whether supplementing pregnant women would prevent this situation. Similarly, iron deficiency could decrease the weight of the newborn, increase the possibility of premature delivery, and increase complications during delivery. In this case, the administration of iron during pregnancy is a strategy that is widely accepted, but for which no controlled studies have been developed [1,11]. Its benefit is debatable for women who do not have an iron deficiency [18].

No single strategy will lead to large improvements, which makes it necessary to seek strategies to improve the nutritional status of pregnant women in general [7,10,11].

The present study aims to summarize evidence on the effects of nutritional deficiencies or excesses on maternal and newborn morbidity. The manuscript includes strategies that may be useful for the different actors involved: medical educators, project administrators, and practitioners.

## 2. Methods

In preparing for this review, PubMed searches using the terms “pregnancy” OR “maternal-fetal health” AND “vitamins” OR “minerals” OR “supplementation” AND “requirement” OR “deficiency nutrients” were performed (Table 1).

In the search process we included the following terms: #1: (“maternal-fetal” [All Fields] AND “health” [MeSH Terms]) OR “Pregnancy” [MeSH Terms] 931,451 results; #2: ((vitamins [MeSH Terms]) OR (minerals [MeSH Terms])) OR (supplemen-tation) 360.554 results; #3: (requirement) OR (deficiency nutrients) 2.166.604 results. The search equation resulting from the combination of terms in PubMed was ((#1) and (#2)) and (#3) 3.099 results. In Table 1, the search process to identify the papers included in this review is illustrated.

The date range for the bibliographic review was December 2019 to July 2021. Specific searches for subtopics included “vitamin A”, “vitamin C”, “vitamin E”, “vitamin D”, “vitamin K”, “vitamin B”, “Folic acid”, and “guidelines”.

All articles were recovered and selected on the basis of the presence/absence of the search criteria. To identify any articles that may have been missed during the literature search, reference lists of candidate articles were also carefully checked. Approximately 210 reports, reviews, and studies were identified through this process. The sources of information consulted were:

- Scientific articles published in official databases (PubMed database, Cochrane Database).
- Scientific books.
- Official web pages (Spanish Society of Gynecology and Obstetrics (SEGO); the American College of Obstetricians and Gynecologists (ACO); Micronutrient Information Center (MIC); Spanish Nutrition Society (SEÑ); Food and Agriculture Organization of the United Nations (FAO); World Health Organization (WHO); International Atomic Energy Agency (IAEA); National Institutes of Health (NIH); American Diabetes Association (ADA)).

Non-English- or Spanish-language articles, articles with very narrow areas of application, and articles without clear scientific evidence were avoided.

## 3. Results

### 3.1. Vitamins

In general, given the physiological characteristics of pregnancy, there is an increase in the demand for vitamins.

#### 3.1.1. Vitamin A

Vitamin A was discovered over a century ago [19] and has been considered a public health priority by the World Health Organization (WHO) for more than 60 years [20]. In humans, this vitamin can be found in three different active forms (retinal, retinol, and retinoic acid) and in a storage form in the liver (retinyl ester) [21]. Vitamin A must be taken in through the diet, since the human body is not able to synthesize this micronutrient. There are two main sources of vitamin A: preformed vitamin A (retinol and retinyl ester) and provitamin A (carotenoids) [21,22,23].

Vitamin A plays an important role in cell division, differentiation, and proliferation, as well as in the development and maturation of organs. Consequently, its deficiency is associated with premature births, intrauterine growth retardation, and low birth weight, as well as an increase in maternal mortality [11]. Vitamin A and related retinoids play an important role in the regulation of immune function and can interfere with the occurrence or worsening of previous or coexisting diseases during pregnancy, childbirth, and the postpartum period.

It is essential to maintain appropriate levels of vitamin A during pregnancy to support the health of the mother and the fetus [24]. The child obtains this vitamin from the mother: via the placenta during gestation and at birth, and via the mammary glands during lactation (breastfeeding). Pregnant women present decreased levels of serum retinol, especially in the third quarter. Due to this, and to the selective placental barrier, newborn hepatic levels of vitamin A are also low to prevent potential teratogenic effects [25].

Vitamin A deficiency in pregnant women is more common when a shortage of vitamin A-rich foods exists, or when there is a case of diabetes mellitus or gestational diabetes [26,27].

There is strong evidence from animal studies that vitamin A deficiency is associated with adverse effects on offspring during the embryonic and postnatal period [28,29,30]. The need for vitamin A in more advanced stages of development is also evident in experimental rodent models. The main target tissues of vitamin A deficiency include the heart; the central nervous system and its derived structures; the circulatory, urogenital, and respiratory systems; and the skull, skeleton, and limbs [29]. 

Studies in humans suggest that low or excessive levels of vitamin A in the diet during pregnancy can result in adverse effects on the fetus [31]. Animal studies involving different species suggest that excess vitamin A might lead to teratogenic effects [32]. Additionally, it has been shown that the type of malformation is influenced by the level of this vitamin and the gestational stage at the time of administration [33].

Vitamin A supplementation also correlates with a reduced risk of infections, due to its relevant function in immune responses and contributing to the host’s defenses [34,35]. Vitamin A deficiency appears to facilitate premature placental abruption and pre-eclampsia. Recent theories on the role of oxidative stress in the pathophysiology of pre-eclampsia have generated great interest in the effect of β-carotene during pregnancy. Free radicals are believed to be toxic elements that negatively affect maternal vascular function. A recent randomized study seems to confirm this theory of oxidative stress as a cause of pre-eclampsia, showing that low levels of vitamins E and C and β-carotene (all of them antioxidant vitamins) are associated with an increased risk of pre-eclampsia. It should be noted that the women participating in this study were recruited due to abnormal uterine arterial Doppler, so the effects of population-based supplementation would be much lower [36].

Other studies document an association between levels of vitamin A in the serum of mothers and the risk of transmitting HIV from mother to child in infected women. According to a systematic review carried out in 2015, supplementing vitamin A during prenatal care did not result in a reduction in maternal or perinatal mortality. However, this review combined data from studies that included diverse populations with different basal levels of vitamin A, with no information regarding vitamin deficiency. Moreover, there was no accurate follow-up with these women. This review concluded that supplementing HIV-infected pregnant women with vitamin A might reduce nocturnal blindness and anemia but has no effect on the vertical transmission of HIV. It was also suggested that this supplementation might lead to a reduction in maternal infection rate. However, as previously discussed, the quality of the data is questionable [33,37,38].

So far, there have been no studies that show unquestionably that a vitamin A supplementation program benefits maternal mortality rates and neonatal weight or reduces preterm deliveries. Administration of vitamin A during pregnancy is needed to maintain the serum retinol levels of women residing in areas where vitamin A deficiency is a public health concern, but we cannot identify strong evidence of a general benefit of vitamin A supplementation during pregnancy [39]. The evidence is not conclusive to support the prescribing of vitamin A supplements during pregnancy.

#### 3.1.2. Vitamin C

The levels of vitamin C are generally lower in pregnant women than in nonpregnant women, probably as a consequence of hemodilution and active transfer of this vitamin to the fetus. The requirement for this vitamin is increased in pregnant and lactating women [40,41]. 

It is known as ascorbic acid, and together with vitamins E and A, it makes up the group of antioxidants [42].

Ascorbic acid deficiency has been associated with an increased risk of infections, premature rupture of membranes, and pre-eclampsia [11].

Randomized, double-blind, and placebo-controlled studies show that vitamin C, like the other antioxidant vitamins, contributes to reducing oxidative stress and consequently to improving the course of pre-eclampsia. Vitamins E and C act in a complementary and synergistic way: vitamin C helps to transform oxidized vitamin E back into its useful form and thus collaborates by restoring vitamin E reserves [43]. In these studies, the proportion of preterm deliveries was higher in the placebo group than in the antioxidant group, supporting the hypothesis that oxidative stress is responsible for the endothelial dysfunction typical of pre-eclampsia. However, before introducing ascorbic acid as a routine procedure in the clinical management of pre-eclampsia or premature rupture of membranes, it would be necessary to carry out a multicenter study with a larger number of patients.

In smokers, it has been proven that it is necessary to increase intake of vitamin C, since their diet usually contains less fruit and vegetable matter. Furthermore, even with adequate vitamin C intake, the serum levels of this vitamin are lower. Tobacco use can lead to a situation of oxidative stress for the pregnant woman and her child [11].

The dietary reference intake has been set at 85 mg per day, 10 mg per day more compared to that of average adult women. In the case of smokers and other high-risk groups, an increase in intake of 50 mg per day is considered necessary compared to nonsmoking adult women [11].

Some studies indicate a moderate increase in the risk of preterm birth in pregnant women with vitamin C supplementation, while an excess could cause “rebound” scurvy in the newborn.

#### 3.1.3. Vitamin E

Vitamin E or tocopherol is an important antioxidant for successful pregnancy. Although vitamin E deficiency is rare in healthy adults, insufficient dietary intake of vitamin E in pregnant women can lead to complications such as pre-eclampsia or premature detachment of the placenta [11,43].

It seems that women with a poorer vitamin E status more often have premature babies, with low birth weight and a higher risk of hemolytic anemia. In preterm infants, it has been associated with bronchopulmonary dysplasia, intraventricular hemorrhage, periventricular leukomalacia, retinopathy of prematurity, and necrotizing enterocolitis.

On the other hand, as the age of the pregnant woman increases, there is a decrease in the levels of vitamin E in serum and later in breastmilk. Therefore, it would be advisable to monitor the nutritional situation in older pregnant women and prescribe supplements [11].

The dietary reference intakes in pregnancy do not differ from that of adult women: these being 15 mg of tocopherol per day in USA and 10 mg in Europe [10,44,45]. 

A meta-analysis was conducted on vitamin E supplementation, involving 21 trials with more than 21,000 women. Overall, the trials were of variable quality, and there were only three studies on vitamin E supplementation, none of which contributed data [43]. The rest of the studies included vitamin C and additional supplements.

There was a reduction in the number of premature placental abruptions in women who received vitamin E supplements in combination with other agents. However, it is not clear whether this result was due to vitamin E or to the other agents used in the supplement [43].

The meta-analysis showed that there may be detrimental effects associated with vitamin E supplementation in pregnancy, as there was an increased risk of abdominal pain and premature rupture of fetal membranes at term in women who received vitamin E supplements in combination with other supplements [43]. The data do not support the administration of vitamin E supplementation in combination with other supplements for the prevention of maternal and neonatal mortality, preterm birth, pre-eclampsia, premature membrane rupture, or poor fetal growth [43].

Ultimately, there is no convincing evidence that vitamin E supplementation in combination with other supplements leads to significant benefits or harms in pregnancy [10,41].

#### 3.1.4. Vitamin D

Vitamin D is also called calciferol, ergocalciferol (D2), or cholecalciferol (D3). The body can synthesize it through sun exposure or acquire it through the diet. It is essential for the maintenance of bone mineralization through the regulation of calcium and phosphorus homeostasis. It also has effects on the immune, endocrine, and cardiovascular systems [46].

The biologically active form of vitamin D (1,25-dihydroxycholecalciferol) circulates in plasma in high concentrations during pregnancy and is essential for the effective deposition of calcium in the fetus [11,42].

Vitamin D deficiency can produce alterations in calcium metabolism, both in the mother and in the fetus, the most frequent being neonatal hypocalcemia and tetany, infantile tooth enamel hypoplasia, and maternal osteomalacia. In the case of neonatal hypocalcemia, its incidence could be reduced by supplying 10 µg per day [11,47].

The correlation between vitamin D deficiency and adverse pregnancy outcomes has been widely studied in recent years. It is theorized that vitamin D deficiency might result in an increased risk of pre-eclampsia, gestational diabetes mellitus, caesarean section, and bacterial vaginosis in pregnancy [48].

Many studies have shown a positive effect of the intake of nutritional supplements on vitamin D concentration in pregnant women [49,50,51,52,53]. However, these studies are very heterogeneous in number and methodology. Hollis et al. compared three different dosages of supplemented vitamin D, namely 400 IU, 2000 IU, and 4000 IU, daily [54]. The group that received 4000 IU of vitamin D reached the highest concentrations [54]. In a subsequent analysis of this randomized controlled trial, the authors suggested that a supplementation dose of 4000 IU of vitamin D enables pregnant women to achieve optimal vitamin D concentrations [55]. This was the first paper to report in an intention-to-treat fashion that supplemented vitamin D decreased some important pregnancy complications.

Evidence also suggests a role of vitamin D in insulin secretion and sensitivity, helping to maintain glucose tolerance [56].

The association between vitamin D deficiency and the incidence of gestational diabetes has been studied by different groups, and diverse conclusions have been derived. One such prospective study investigated vitamin D levels at week 16 of pregnancy in a group of 57 women who later experienced gestational diabetes, and 114 women who did not [57].

More research needs to be done to fully understand the exact mechanism of altered vitamin D metabolism in patients with pre-eclampsia and hypertensive disorders [58]. Many observational studies found a significant association between lower vitamin D concentrations and higher risk of pre-eclampsia.

In a comparative study performed by Baker et al. [59], which involved a group of American mixed-ethnicity pregnant women, a high incidence of vitamin D deficiency was recognized. Other investigators also reported lower vitamin D concentrations in women with pre-eclampsia or those who developed pre-eclampsia later in pregnancy in comparison to those who did not [60,61,62,63]. On the other hand, some observational studies found no difference in vitamin D concentrations in women with pre-eclampsia compared with normotensive women. A study of 221 mixed-ethnicity Canadian women measured vitamin D concentrations at the 15th and the 20th week of pregnancy [64]. No difference was observed between the three groups in terms of the development of pre-eclampsia later in pregnancy. However, only 28 patients developed pre-eclampsia, so the low number of participants might influence the results.

Calcium supplementation is recommended for high-risk women with low-calcium diet. Vitamin D plays a role in calcium homeostasis and helps maintain appropriate levels of calcium in the body, which is inversely associated with blood pressure. Therefore, it is recommended that pregnant women supplement their diet with vitamin D [65].

In addition, vitamin D is associated with preterm delivery, since it influences the processes of inflammation and immunomodulation [66]. 

Results from several observational studies suggest that there is no association between maternal vitamin D levels and preterm labor [67,68,69]. Nonetheless, some of these studies included participants with specific conditions such as previous history of preterm birth [70], twin gestation [66] or high risk of pre-eclampsia [71]. Hence, these results must be interpreted with caution. 

The influence of vitamin D on preterm birth was investigated in a mixed-ethnicity American supplementation study, and an inverse association was found [72]. When analyzing maternal vitamin D concentrations at the time of labor, a 62% lower risk of preterm delivery was reported in women with vitamin D concentrations higher than 40 ng/mL, compared to those below 20 ng/mL. Similar results were reported by Wagner et al., where vitamin D concentrations were measured in mixed-ethnicity American women within six weeks of delivery. A 57% lower risk of preterm labor was observed in women with vitamin D levels above 40 ng/mL, compared to concentrations lower than 20 ng/mL [73].

The association between vitamin D status and autism spectrum disorder (ASD) is well-investigated but remains to be elucidated. The detection of and appropriate intervention addressing vitamin D deficiency in ASD patients and pregnant and lactating women have clinical and public significance [74]. 

The deficiency is more frequent during the winter, in women who live in countries or areas with little sun exposure, and in those who follow vegetarian or vegan diets. Therefore, in these cases it would be necessary to pay more attention and try to ensure an adequate intake of vitamin D through the diet (e.g., through milk, fortified dairy products, and fatty fish) or through nutritional supplements if necessary [11,47].

The need to improve maternal vitamin D status is of special importance in developing countries in order to support the best maternal and child health outcomes [75].

#### 3.1.5. Vitamin K

Vitamin K is an essential cofactor for the carboxylation of glutamic acid residues in many vitamin K-dependent proteins that are involved in blood coagulation, bone metabolism, prevention of vessel mineralization, and regulation of various cellular functions. Deficiency of vitamin K can be critical for pregnant women and especially newborns, possibly resulting in hemorrhage. Prothrombin requires vitamin K for blood coagulation [76].

Deficiency of vitamin K can worsen when certain drugs such as heparin and carbamazepine are consumed during pregnancy, because the drugs can impede women’s metabolism of vitamin K [76,77]. Additionally, the fetus can be affected by exposure to drugs in utero, in which case onset of coumarin embryopathy (CE) is possible [78].

The recommended intake level of vitamin K is set to 90 µg per day for women. There are insufficient data to establish differentiated recommended intakes during pregnancy [10,13]. Regular diets contain a high contribution of this vitamin in relation to the recommended intakes of nonpregnant women, so there is no need for specific recommendations. Although vitamin K supplementation is not necessary in normal pregnancy, deficiency may occur in women with epilepsy and other impaired conditions [78,79,80,81].

#### 3.1.6. Vitamin B6

Vitamin B6 or pyridoxine is involved in the formation of neurotransmitters, in the synthesis of the heme group, and in the formation of myelin. It is essential for the development of the nervous and cognitive system, and its deficit usually manifests itself in the form of neurological symptoms, skin lesions, or anemia. In addition, the presence of this vitamin reduces homocysteine levels in the blood, thereby reducing cardiovascular risk [11,42].

Studies on pyridoxine supplementation during pregnancy have shown that it may be effective at reducing maternal nausea and vomiting and the risk of orofacial clefts (cleft lip, palate) and cardiac malformations in the newborn. Better Apgar scores in the first minute and greater weight have also been observed in neonates.

In 2007, Ronnenberg et al. proposed that a low maternal concentration of vitamin B6 before pregnancy can have effects on the early stages of pregnancy. Taking into account the association between vitamin B6 deficiency and the alteration of the enzymes that affect the structural integrity of the arterial walls, it is assumed that such a deficiency affects implantation and early placental development. Therefore, evaluating the hematological levels of this vitamin before conception would be very useful, since they probably affect the periconceptional period [82].

Since the plasma concentrations of pyridoxal phosphate, an active metabolite of vitamin B6, are lower in pregnant women (not so in fetuses, whose levels are high), high intake (more than 10 mg per day) is needed to prevent this plasma decline. This dose cannot be achieved through the diet, so an additional intake of 0.6 mg of vitamin B6 is needed to achieve an intake of 1.9 mg per day [11].

Vitamin B6 deficiency may account for some types of anemia in pregnancy, as demonstrated by a study of 56 anemic Japanese pregnant women who were unresponsive to iron supplementation but responded to vitamin B6 therapy [83].

The potential benefits of vitamin B6 supplementation are a reduction in nausea and vomiting, improvement in dental health, and treatment of some cases of anemia. In a meta-analysis based on three small studies, vitamin B6 supplementation had a significantly positive effect on birth weight (d = 217 g [95% confidence interval (CI) 130, 304]) [84].

A Cochrane review concluded that there is a lack of consistent evidence that this therapy is effective at reducing symptoms during early pregnancy [85]. Doses of 50–510° mg/day taken during the first trimester have not been associated with adverse fetal outcomes [86,87].

#### 3.1.7. Vitamin B3

Vitamin B3 (or niacin) deficiency causes pellagra, whose classic triad of symptoms is dermatitis, diarrhea, and dementia.

During pregnancy, there is a greater capacity to convert tryptophan to niacin, which is related to a higher rate of estrogen. The increase in energy requirements during pregnancy represents a reference dietary intake of 18 mg per day of niacin equivalents, compared to the 14 mg per day recommended for adult women. However, its supplementation is not recommended, since there is no evidence that its deficit or excess has adverse effects during pregnancy [42].

A recent, prospective, exploratory pilot study was conducted on miscarriage, involving 24 women who were <14 weeks pregnant. Neither niacin intake (*p* = 0.24) nor urinary vitamin B3, measured as the 1-methyl-5-carboxylamide-2-pyridone/N-1-methylnicotinamide (2-pyr/MNA) ratio (*p* = 1.00), predicted miscarriage. However, the difference in mean 2-pyr/MNA ratios between women who miscarried and controls suggests there may be a threshold niacin level that protects against miscarriage; this warrants further investigation [88].

#### 3.1.8. Vitamin B1 and B2

Vitamins B1 and B2 are essential for fetal growth, and their levels during pregnancy have been related to birth weight. Thiamine is vital for muscle, nerve, and bone development in babies. Routine supplementation is not recommended for pregnant women with normal nutrition.

Vitamin B1 or thiamine deficiency is related to the appearance of beriberi and Wernicke’s encephalopathy, a complication of hyperemesis gravidarum [89]. Wernicke’s encephalopathy is a neurological disease caused by a deficiency of vitamin B1 or thiamine. It occurs in any situation that prevents the absorption of the vitamin, such as hyperemesis gravidarum. It manifests with the classic triad of ataxia, ophthalmoplegia, and mental confusion, although sometimes the symptoms are very nonspecific. If the disease is untreated, it can cause irreversible damage. Diagnosis and early treatment are essential to avoid both maternal and fetal sequelae [90,91,92,93]. Wernicke’s encephalopathy is reversible if treated with a timely dose of parenteral thiamine [94].

Vitamin B2 or riboflavin acts as a coenzyme in energy utilization and as a cofactor of glutathione reductase, so it can be considered an indirect antioxidant. Its deficiency causes a clinical syndrome characterized by cheilosis, stomatitis, glossitis, keratitis, ocular disorders, and seborrheic dermatitis [42].

Maternal or fetal complications associated with low levels have not been demonstrated, so routine supplementation during pregnancy is not recommended. However, the recommended dose for both vitamins during pregnancy is 1.4 mg per day [11].

#### 3.1.9. Folic Acid and Vitamin B12

Folic acid belongs to the group known as folates, a set of essential nutrients that participate in the synthesis of DNA and proteins. For this reason, it is essential in periods of high metabolic activity, such as pregnancy, where there is a high rate of cell replication [36].

It is known that there is a close relationship between folic acid deficiency and neural tube defects (NTD), produced as a consequence of a failure in neural tube fusion during days 21 and 27 of embryonic life. They can manifest in the brain in the form of anencephaly or encephalocele (incompatible with life) or in the spinal column as spina bifida [4,9,11,95,96,97].

Numerous epidemiological studies have demonstrated such a relationship. Smithells et al. were the first to document it in 1976 [98]. Subsequently, Laurence et al. demonstrated that supplementation with 0.4 mg of folic acid per day in pregnant women was able to reduce the incidence of NTDs [99].

The most significant clinical trial was conducted by the UK’s Medical Research Council. This study showed that supplementation with 4 mg of folic acid per day in high-risk pregnant women with a previous child affected by NTD decreased the recurrence of the defect by 72%. The levels of folic acid should be elevated during the period of conception and up to 30 days later, which is when the closure of the neural tube ends [100].

In addition, there are studies that propose that the joint intake of folic acid and vitamin B12 (metabolically related) contributes to reducing the risk of nongenetic congenital malformations, including NTDs [101,102]. Raghavan et al. examined the association of multivitamin supplementation during pregnancy and biomarker measures of maternal plasma folate and vitamin B12 levels at birth with the child’s Autism Spectrum Disorder (ASD) risk, showing that extremely high maternal plasma folate and B12 levels at birth were associated with ASD risk [103].

A deficiency of folic acid (and also of vitamin B6 and B12) gives rise to an increase in homocysteine in the blood, a nonessential amino acid that, in high concentrations, has neurotoxic, vasculotoxic, and therefore teratogenic effects. It is believed that this is the mechanism by which NTDs and other pathologies mediated by placental vasculopathy such as spontaneous abortion, premature placental abruption, and pre-eclampsia arise [104,105]. 

Taking folic acid has also been shown to decrease the rate of cleft lip and congenital heart disease (as has vitamin B6). Moreover, a recent study suggested that folic acid supplementation during pregnancy reduces the risk of childhood acute lymphoblastic leukemia by 60% [106].

A recent systematic review and meta-analysis of case-control studies determined the effects of maternal folic acid consumption on the risk of childhood cancer. Maternal folic acid supplementation was found to have a protective effect against childhood acute lymphoblastic leukemia [107].

The European Surveillance of Congenital Anomalies (EUROCAT) developed a report that analyzes the official recommendations of 17 countries and the strategies used to reduce the incidence of NTDs, drawing the following conclusions [108]:

- There is evidence that most NTDs are preventable by increased folate intake, and the benefit probably extends to other birth defects as well.
- The development of informative and educational programs for the population is important.
- The daily intake of folates in the diet should be increased and supplemented with folic acid before conception.
- Foods fortified with folic acid, properly identified and directed to the target population, should be introduced.

International guidelines (i.e., from the World Health Organization) currently recommend supplementation of folic acid (0.4 mg/day) during the whole pregnancy for the purpose of improving pregnancy outcomes and reducing maternal anemia in pregnancy [109].

In Spain, the intake of a supplement of 0.4 mg of folic acid per day is recommended for all women who are planning a pregnancy and 4 mg per day for those with a history of NTD. This supplementation should be started at least one month before conception and maintained at least until the end of the first trimester [110,111]. Doses greater than 1 mg per day could mask the neurological alterations of pernicious anemia; however, as it is a rare disease, the benefits for women with a history of NTD outweigh the risks.

Intake of folates together with multivitamin complexes during pregnancy (as long as they do not contain fat-soluble vitamins above the daily doses recommended) reduces the incidence of cardiac, urinary, orofacial, and limb malformations and pyloric stenosis. Based on these observations, the Society of Obstetrics and Gynecology of Canada recommends using different strategies in the primary prevention of NTD, as well as other congenital malformations, depending on the characteristics of each woman [112,113].

On the other hand, vitamin B12 or cobalamin is a water-soluble vitamin essential for the normal operation of the brain and the nervous system, and for the formation of blood and several proteins. Its deficit mainly produces megaloblastic anemia and neuropathy, with diffuse and progressive demyelination [36].

Neurological involvement is related to the lack of methyl groups as a consequence of the inability to synthesize methionine and S-adenosylmethionine or to eliminate the homocysteine toxic to the brain. Let us remember that vitamin B12 deficiency also produces an increase in homocysteine levels (neurotoxic, vasculotoxic and teratogenic amino acids), which is why it is also related to congenital malformations such as neural tube defects and even arteriosclerosis, maternal obesity, and dyslipidemia [114,115,116].

Recommendations for vitamin B12 are generally 2 µg per day [11]. It should not be used in myeloproliferative conditions, especially in the case of leukemia. In any case, no cases of toxicity due to overdose have been described with intake of up to 1000 µg [117].

Table 2 summarizes the recommended daily dose of vitamins in nonpregnant and pregnant women.

### 3.2. Minerals

#### 3.2.1. Calcium, Phosphorus, and Magnesium

Calcium is the most abundant mineral in the human body. It is essential for bone maintenance, nerve transmission, neuromuscular excitability, smooth muscle contraction, blood clotting, and enzyme activation [42].

During pregnancy, calcium metabolism undergoes a series of changes in order to maintain adequate levels in maternal plasma and bone to facilitate its maternal–fetal contribution:

- Its absorption increases dramatically in the second and third trimesters and is greater when the calcium contribution is lower. The responsible hormone is a peptide similar to parathyroid hormone (PTH), recognized by the same receptors and synthesized by the fetus. On the other hand, vitamin D doubles its levels in the pregnant woman, also allowing for increased absorption of calcium [118].
- Maternal bone turnover appears to decrease in studies monitoring markers of bone formation and resorption [119,120].
- Physiological hypercalciuria occurs as a result of increased absorption [121].

Multiple studies have linked calcium deficiency to the development of pre-eclampsia: generalized microangiopathy characterized by the appearance of arterial hypertension and proteinuria from the 20th week of gestation in a previously healthy woman. Furthermore, this pathology is a frequent cause of prematurity [9,122,123].

Most epidemiological studies on calcium supplementation during pregnancy demonstrate an inverse relationship between calcium intake in diet and the incidence of hypertensive disease during the pregnancy. In the latest Cochrane review [124], high-dose calcium supplementation (≥1 g/day) reduced the risk of pre-eclampsia and preterm birth, particularly for women with low-calcium diets. However, the limited evidence on low-dose calcium supplementation suggests a reduction in pre-eclampsia, hypertension, and admission to neonatal high care, but this needs to be confirmed by larger, high-quality trials.

For this reason, universal calcium supplementation is not recommended during pregnancy, and the recommended dose is equal to that of a nonpregnant woman of reproductive age: 1000 mg per day of calcium. However, supplementation is recommended for high-risk women [9]:

- Pregnant women from developing countries.
- Pregnant women under 18 years: 1300 mg a day.
- Subgroups with low calcium intake (less than 600 mg per day).
- Pregnant women with high risk of pre-eclampsia.

In any case, diet and pharmacological supplementation should not provide more than 2500 mg of calcium per day, since an excess can cause hypercalcemia, kidney stones, alkalosis, and kidney failure [11].

Phosphorus is a fundamental component of nucleic acids and cell membranes. It is involved in the transport and production of energy in the form of ATP and in the acid–base balance, as well as stimulating bone mineralization and activating multiple metabolic pathways such as glycolysis and gluconeogenesis. Its metabolism is closely related to that of calcium. It can be acquired through a large number of food products, so its dietary deficiency is rare, and routine supplementation during pregnancy is not recommended [42].

Magnesium is an essential mineral necessary for the regulation of body temperature, the synthesis of proteins and nucleic acids, and for the maintenance of the electrical potentials of nerve and muscle cells.

Studies have suggested that magnesium supplementation during pregnancy could reduce pre-eclampsia and increase birth weight.

However, in a Cochrane review of the Pregnancy and Childbirth Register that analyzed 10 randomized trials including 9090 women and their infants, no significant differences in the risk of perinatal mortality were observed when comparing the group of infants of mothers who received magnesium during pregnancy and the group of infants of mothers who did not receive magnesium. Furthermore, magnesium supplementation did not reduce the risk of infants being small for gestational age, nor did it reduce the risk of pre-eclampsia in mothers. Therefore, no convincing evidence was found that magnesium supplementation during pregnancy has beneficial effects [125].

Several studies suggest that the risk of gestational hypertension is related to changes in magnesium (Mg) homeostasis [126]. In a randomized controlled trial, Bullarbo et al. investigated the effect of magnesium supplementation in healthy pregnant women for prevention of blood pressure increase. They concluded that magnesium supplementation in healthy first-time pregnant women is not to be recommended for the prevention of blood pressure increase [127].

#### 3.2.2. Iodine

Iodine status is a global health concern, particularly in developing countries, and emphasis should be placed on diagnosis and correction at the community level rather than the individual due to the high impact on child neurological development and pregnancy outcomes [128,129].

Iodine deficiency is responsible for multiple pathologies: endemic goiter, recurrent miscarriages, growth retardation in children and adolescents, mental retardation, and cretinism. Its most serious consequence is alterations in the brain and neurological development of the fetus, which are irreversible at birth [11].

The amount of thyroxine (T4) circulating in the maternal blood determines the optimal development of the fetal cerebral cortex, especially in the first half of pregnancy. In the first trimester, there is a physiological increase in the concentration of circulating T4, which subsequently decreases. In the second half of pregnancy, the fetal thyroid begins to secrete its own thyroid hormones, but in insufficient quantity, so the maternal contribution continues to be essential [130]. 

A recent study showed a significant correlation between children’s intelligence quotient (IQ) and the concentration of free T4 in the maternal plasma during the first trimester of pregnancy, but not later on. Among the children of women with low thyroxine levels, there were also a high number of cases of attention deficit and hyperactivity, as well as problems with psychomotor development and lower values of IQ [131]. 

The World Health Organization (WHO) has defined iodine deficiency as the leading cause, after extreme starvation, of preventable mental retardation and cerebral palsy in the world [132]. For this reason:

- The use of iodized salt is an essential and urgent measure to correct the iodine deficiency in the general population. It is a global priority in public health.
- In pregnant women, this measure is insufficient, since higher daily doses of iodine are needed (200 µg more) that cannot be achieved only through salt intake. Therefore, in addition to the consumption of iodized salt, the use of supplements in the form of potassium iodide is necessary.

Despite these measures, data from various epidemiological studies show that most women in Europe have iodine deficiency during pregnancy, and only 13–50% receive supplements of the mineral during pregnancy [130].

As with folates, it is recommended to start the supplementation before pregnancy and maintain it during breastfeeding, since breastmilk is the only source of iodine for the child at a time when brain development continues to require thyroid hormones [11].

Excessive iodine intake produces an increased risk of autoimmune thyroiditis or hyperthyroidism in the mother and neonatal hypothyroidism. However, the use of these supplements does not pose any risk, because the amounts used, even adding the usual consumption of iodized salt and marine fish, are too low to cause problems [130].

#### 3.2.3. Iron

Iron is an essential trace element that is part of hemoglobin and therefore participates in the transport of oxygen.

Iron content in the human body is carefully regulated and is normally maintained at about 40 mg/kg in women and about 50 mg/kg in men. Since humans are unable to excrete excess iron in a regulated manner, iron balance is controlled at the levels of iron absorption by enterocytes in the duodenum, and of iron mobilization from the liver parenchyma and macrophages [133].

Iron needs increase in pregnant women; in fact, iron deficiency anemia is the most common nutritional deficiency among pregnant women (its prevalence increases to 15–20%). As a compensatory mechanism, it seems to increase the efficiency of its absorption, although it is difficult to determine if this is sufficient to cover the needs during pregnancy [134].

The effect of iron deficiency on erythrocyte production occurs in the context of a phenomenon referred to as physiological anemia of pregnancy, which is conserved in mammals [135,136].

Iron is necessary for the placenta, uterine enlargement, increased red blood cell synthesis, and fetal growth. The requirement of maternal iron transfer for a normal single pregnancy carried to term has been estimated at 500–800 mg [137]. The average demand for absorbed iron over the course of pregnancy is around 4.4 mg/day, being lower in early pregnancy (0.8 mg/day) than in late pregnancy (7.5 mg/day) [138]. Iron deficiency can impair cognitive development in early childhood [139], so it is of interest from a public health perspective to avoid this complication by intervening during pregnancy. However, it is unclear whether maternal iron supplements can reverse the effects of maternal iron deficiency during pregnancy on neural development [140,141]. In a study by Milman et al. on healthy pregnant women, a consistent correlation between iron supplementation and the 5th percentile Hb value was observed, by comparing a test group supplemented with 66 mg elemental iron/day and a control group receiving a placebo. These differences gradually increase throughout the pregnancy, being smaller in the first trimester (0.1 mg/dL Hb higher) and increasing in the second (0.1–0.4 mg/dL Hb higher) and third trimesters (0.3–0.9 mg/dL Hb higher) and reaching the highest value in the postpartum period (1 mg/dL Hb higher) [142]. The smaller difference in the first trimester might be due to a high incidence of iron deficiency or marginal iron stores in the test and control groups, and the increasing differences likely reflect the increasing iron requirements that are met by the supplemented group of women but not by the placebo group. Currently, neither the US Preventive Services Task Force nor the American College of Obstetricians and Gynecologists (ACOG) takes a position on routine iron supplementation in pregnancy [143,144,145,146]. UK guidelines do not recommend this supplementation. In keeping with the UK and ACOG guidelines, the investigation of an etiology of anemia would be feasible if Hb levels were below 11.0 g/dL in the first and third trimesters and below 10.5 g/dL in the second trimester (ACOG), or below 11.0 g/dL in the first trimester and below 10.5 g/dL in subsequent trimesters (UK). At this point, there is a shift in focus from supplementation to treatment of iron deficiency anemia. The objective of the treatment is to correct the anemia and replenish iron stores. Such treatment would have to account for the transfer of 500–800 mg of iron to the newborn and maintain the Hb/Hct balance in the mother, while also repleting iron stores. One suggested approach consists of providing 60–100 mg of elemental iron per day to pregnant women.

Two main iron supplementation approaches exist for nonanemic pregnant women. One well-described approach is based on selective supplementation, given by the estimation of iron concentrations by serum ferritin. When the serum ferritin value rises above 70 μg/L, the iron concentration is considered sufficient to support pregnancy, so no iron supplements are required. However, when serum ferritin drops below 30 μg/L, the patient is given 80–100 mg elemental iron/day orally to replace the absent or nearly absent iron stores. Patients whose ferritin levels are between these two thresholds are treated with a low iron dose of 30–40 mg/day [147,148]. A recent systematic review proposed that intermittent iron supplementation during pregnancy (two or three times per week) is as effective as daily supplementation, decreases side effects, and probably results in higher compliance [149]. 

#### 3.2.4. Zinc

Zinc is an essential nutrient that maintains the activity of a great variety of enzymes in different metabolic pathways, and thus is involved in vital functions for cells, such as mitosis, DNA synthesis, protein synthesis, and genetic expression and activation [11].

Given its functions, it is logical that its requirements increase during pregnancy, requiring the intake of at least 11 mg per day [11]. However, the literature data on the influence of zinc supplementation on the course of pregnancy and fetal development are inconclusive [150].

It is considered that around 82% of pregnant women worldwide do not ingest zinc in sufficient quantities, which can have consequences for the health of the fetus [4,100].

- When the zinc deficit is moderate, the risk of premature rupture of membranes, premature delivery, and low birth weight increases. In addition, alterations in immune development can occur.
- If the deficit is severe, congenital malformations could occur: palatal, cardiac, urological, skeletal, and brain defects.

Thus, zinc supplementation has been associated with an increase in birth weight and a decrease in perinatal complications, although these facts have not been proven in randomized trials [151].

There are studies that link zinc deficiency with the appearance of pre-eclampsia, postpartum hemorrhage, and even spontaneous abortions [36]. However, data from the Cochrane database of systematic reviews suggest that zinc supplementation may result in little or no difference in terms of reducing the number of preterm births, the risk of stillbirth, or the number of perinatal deaths. It is unclear whether zinc supplementation reduces the rate of neonatal death. Finally, in terms of other birth outcomes, zinc supplementation may make little or no difference to mean birth weight, i.e., to the risk of low birth weight and small-for-gestational-age babies when compared to the placebo or no-zinc supplementation [152].

The disagreement between studies could be a consequence of nutritional differences between the populations targeted for the intervention. Thus, there is not enough evidence that zinc supplementation during pregnancy results in improvements in maternal or neonatal outcomes. 

It is important to consider that, during pregnancy, there are zinc homeostatic adjustments that sufficiently improve zinc utilization to fulfill the increased zinc needs; therefore, the elevated requirement for zinc by supplementation is compensated by better absorption efficiency in pregnant women [153].

#### 3.2.5. Selenium

Selenium is a powerful antioxidant that the body uses in defense against free radicals. Over the past few years, numerous clinical papers have been published that debate selenium supplementation during pregnancy [154,155].

The pregnant woman will be more susceptible to oxidative stress due to the physiological changes she undergoes and the activity of the placenta. For this reason, selenium increases progressively during pregnancy, reaching its maximum in the second trimester.

Several studies have shown that, in certain complications associated with pregnancy, such as gestational diabetes or pre-eclampsia, there is an increase in oxidative stress. Faced with this situation, the body responds by enhancing its antioxidant mechanisms, some of which lie in the diet [156,157]. Selenium deficiency is also associated with an increased risk of miscarriage.

Selenium is one of the selenoproteins, which are crucial for thyroid hormone synthesis and immune response regulation. Selenium’s role in the physiology and pathophysiology of thyroid function is well-known. In the brain, it takes part in antioxidant processes as an essential component of a number of enzymes [157]. Selenium also plays a crucial role in the regulation of the immune response.

Is selenium supplementation justified in clinical practice in pregnant and childbearing women with autoimmune thyroid diseases? It has been hypothesized that since selenium may act as an anti-inflammatory agent in autoimmune thyroiditis, women with this condition could potentially benefit from selenium supplementation [154,158]. 

The recommended selenium intake for pregnant women is about 60 mg per day. This quantity can be acquired through a balanced diet containing meat and organ meats, foods of marine origin, and vegetables (in the latter case, the selenium content will depend on the mineral content in the cultivation soils). However, before the drawing up of selenium supplementation recommendations, conclusive results are required, and we need to be aware of the possible toxic effects of selenium [159].

#### 3.2.6. Copper

The role of copper in the rupture of membranes and pregnancy outcomes has been studied. Kashanian et al. found that copper supplementation during pregnancy has no influence on the rupture of membranes during pregnancy, but improves the mood status of some women [160].

### 3.3. Fatty Acids ω3

Eicosapentaenoic acid (EPA, C20:5) and docosahexaenoic acid (DHA, C22:6) are omega-3 polyunsaturated fatty acids that are well-studied in humans [161].

Because they can be synthesized in the body from their precursor, alpha-linolenic acid (ALA, C18:3), EPA and DHA are not considered essential fatty acids. However, inefficient conversion of ALA to EPA and DHA [162,163] has led to recommendations to include food and dietary supplements as sources of EPA and DHA.

Fatty acids are essential components of cell membranes and precursors of prostaglandins, thromboxanes, leukotrienes, and other essential eicosanoids in the regulation of blood coagulation, the immune response, and inflammatory processes; these are of great importance for the development of the placenta and the fetus. In many countries, pregnant women or women of child-bearing age rarely consume foods suitable as sources of long-chain Omega-3 fatty acids eicosapentaenoic acid (EPA) and docosahexaenoic acid (DHA) [164,165,166,167].

**Figure 2:**
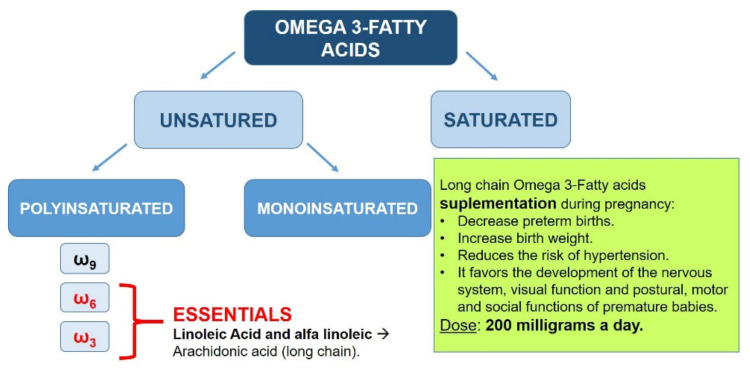
Importance of long-chain fatty acids during pregnancy.

During pregnancy, the plasma phospholipid concentration increases by more than 50%, as a consequence of the hyperlipidemia associated with pregnancy. However, the levels of essential fatty acids and long-chain fatty acids in maternal plasma progressively decrease. Many expert scientific organizations recommend that pregnant women consume an extra 200 mg/day of DHA—for example, in the form of fatty fish from the sea once a week. Increased intake of long-chain fatty acids during pregnancy has been linked with (Figure 2) [168]:

- Lower probability of preterm delivery.
- Greater weight of the newborn.
- Decreased risk of developing hypertension.
- Greater development of the nervous system and visual function.
- Optimization of postural, motor, and social functions of premature infants.

A reduction of premature birth before week 34, inconsistent results with respect to the cognitive development of the child, and data suggesting a reduction of atopic diseases are usually cited in support of the recommendations [167,168,169,170].

Supplementing omega-3 fatty acid in pregnancy is recommended by a number of scientific societies, but not all [168,169,170,171,172].

Evidence of reduced levels of perinatal mortality, premature birth, and complications for the mother were studied in a recent pertinent Cochrane meta-analysis of a total of 70 randomized intervention trials with a total of 19,927 participants. It demonstrated a reduction in premature birth before week 34; in addition, fewer children had low birth weight, and fewer newborns required medical attention. Perinatal mortality was not significantly reduced; pre-eclampsia might have been reduced, corresponding to epidemiologic data. An improvement in postnatal depression was not found, nor were parameters of cognition improved in the child. In the majority of trials, the doses of EPA and DHA used were considerably lower than 1 g/day [173].

While a recent meta-analysis of pertinent intervention trials found no effect of EPA and DHA during pregnancy on postpartum depression, a systematic review saw positive effects [174,175].

In many countries, intake of EPA and DHA by pregnant women or by women with child-bearing potential is low, resulting in low levels of EPA and DHA. This results in premature birth and many other health issues for the mother and child. While it is recommended that pregnant women increase their intake of DHA by 200 mg/day, the correlation between intake and resulting levels is poor, and many pregnant women have a low Omega-3 Index despite following the recommendations. Based on current evidence, von Schacky suggested that a target range for the Omega-3 Index of 8–11% might pertain to pregnancy and lactation [167].

### 3.4. Nutrition for the Prevention and Control of Pathologies Associated with Pregnancy

#### 3.4.1. Pre-Eclampsia

Pre-eclampsia is a multisystemic disease—progressive and of unknown cause—specific to pregnancy, since there must be a pregnancy for it to develop and it disappears at the end of pregnancy [176]. Pre-eclampsia occurs in ~3% of pregnancies and negatively impacts both maternal and fetal health outcomes. The American College of Obstetricians and Gynecologists defines pre-eclampsia as a systolic blood pressure of ≥140 mm Hg or diastolic blood pressure ≥90 mm Hg on two occasions at least 4 h apart, which co-occurs with proteinuria, thrombocytopenia, renal insufficiency, impaired liver function, pulmonary edema, or cerebral or visual symptoms after 20 weeks of gestation [177].

Delivery of nutrients from the mother to the fetus depends on placental vasculature. Placental vascular development requires remodeling of the uterine spiral arteries from narrow vessels to wider, low-resistance vessels [178]. Many studies associate pre-eclampsia with a decrease in uterine blood flow, which leads to impaired fetal nutrition and could also trigger a generalized vasospasm that would affect the rest of the organs and end up producing arterial hypertension [176]. Impairment of vascular remodeling during early gestation is thought to result in later development of pre-eclampsia. Proposed mechanisms leading to pre-eclampsia include impairment of endothelial function and a maternal immune response to the invading trophoblast cells that remodel the spiral arteries [178].

**Figure 3:**
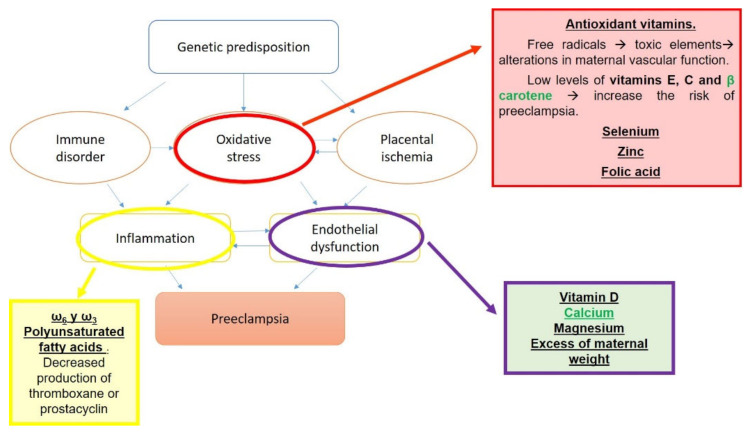
Risk factors for pre-eclampsia.

Multiple risk factors have been proposed for the development of pre-eclampsia (Figure 3). These risk factors can be classified as maternal and environmental, the latter being the focus in this case, since nutrition plays a key role [176,179].

Defects in the development of tolerance and activation of a pro-inflammatory response may be contributing factors to the development of pre-eclampsia [180].

The influence of nutrition on pre-eclampsia has been studied from various perspectives [181].

Omega-3 fatty acids, and particularly DHA, are critical to the structure and function of developing nervous system cells. Several case-controlled studies have demonstrated that, during the third trimester or postpartum, blood levels of omega-3 fatty acids are reduced in mothers with pre-eclampsia compared to normal pregnancy [182].

Kulkarni et al. found that, at delivery, plasma DHA levels were reduced in pre-eclamptic vs. normotensive pregnancy, and this difference was also reflected in infant cord blood samples [183]. 

Regardless of the recommendations from various authorities on the consumption of omega-3 fatty acids, 95% of pregnant women and women of child-bearing age do not consume enough [184]. In cases of pre-eclampsia, the transfer of such fatty acids from the placenta to the cord blood and fetus is reduced [185,186].

Malnutrition is generally accompanied by anemia, which means a deficit in the uptake and transport of oxygen, which can cause hypoxia of the trophoblast. On the other hand, in malnutrition there is also a deficiency of several micronutrients, such as calcium, magnesium, zinc, selenium, and folic acid, the lack of or decrease in which has been related to the appearance of pre-eclampsia [187,188,189].

It seems that excess maternal weight also increases the risk of pre-eclampsia. It has also been observed that women with this disorder have low protein reserves (probably due to proteinuria), which is why it has been suggested that the consumption of an appropriate amount of protein prevents pre-eclampsia [179]. Furthermore, adipocytes secrete cytokines, especially tumor necrosis factor a (TNFa), which cause vascular damage, which worsens oxidative stress, a phenomenon that is also involved in the development of pre-eclampsia [190,191,192].

For its prevention and treatment from a nutritional point of view, it is thought that salt restriction is beneficial, based on the physiological increase in the renin–angiotensin–aldosterone axis in pregnancy and the loss of resistance to the pressor effects of angiotensin II that occurs in pregnant women who are going to develop pre-eclampsia. However, sodium restriction has failed to significantly modify blood pressure, weight gain, or proteinuria. The same can be said of diuretics (contraindicated in pregnancy) and calorie restriction in pregnant women with large weight gain [179].

Interest in maternal supplementation with polyunsaturated fatty acids (PUFA) is growing. Linoleic polyunsaturated fatty acid supplementation has been shown to lower blood pressure and prolong pregnancy in patients with pre-eclampsia. Absence of pre-eclampsia has also been described in patients receiving ω6 and ω3 polyunsaturated fatty acids through parenteral alimentation. In fact, a possible effect of polyunsaturated fatty acids on thromboxane or prostacyclin production has been postulated, the alterations of which are also involved in the pathogenesis of the disease [193].

An association between calcium deficiency and the development of pre-eclampsia has already been discussed. Multiple authors have studied this relationship from different points of view [194]. Epidemiological studies in pregnant women find an inverse relationship between calcium in the diet and pregnancy-induced hypertension [195].

In the Cochrane meta-analysis, 27 studies, with 28,492 pregnant women, were included. The results showed that calcium supplementation was associated with a lower incidence of pre-eclampsia (RR 0.51, 95% CI: 0.40 to 0.64) and gestational hypertension (RR 0.70, 95% CI: 0.60 to 0.82). Subanalyses revealed that a high dose (1.2–2 g/day), moderate dose (0.6–1.2 g/day), or low dose (<0.6 g/day) of a calcium supplement could reduce the risk of pre-eclampsia. For gestational hypertension, only the high-dose and moderate-dose groups were associated with a reduced risk of gestational hypertension. This study indicated that calcium supplementation might decrease the risk of pre-eclampsia and gestational hypertension. However, we could not draw a conclusion as to which dose (high, moderate, or low) was the most protective, as we were unable to directly compare the effects of different doses [196]. 

From all these studies, it can be deduced that adequate calcium intake seems to decrease the generalized vasopressor response that occurs in pre-eclampsia and consequently improves microcirculation. This reduces the possibility of suffering from high blood pressure during pregnancy. 

It is postulated that the interaction between calcium and magnesium may be the real mechanism leading to pre-eclampsia. Magnesium has essential functions in body temperature regulation, nucleic acid and protein synthesis, and maintenance of nerve and muscle cell electrical potentials. The intake of magnesium supplements during pregnancy would presumably reduce fetal growth restriction and pre-eclampsia and increase birth weight. Given the functions of magnesium (described in previous sections: control of intracellular calcium, central vascular tone, and nerve conductivity), it is logical to think of hypomagnesemia as a precipitating factor for pre-eclampsia. Magnesium sulfate has been widely used as the non-nutritional drug of choice, even compared to phenytoin, in the treatment of seizures in eclamptic women [125]. However, the review from the Cochrane Pregnancy and Childbirth Group’s Trials Register showed that there is not enough high-quality evidence to show that dietary magnesium supplementation during pregnancy is beneficial.

The results obtained in multiple studies are contradictory, since an association between hypermagnesemia and pre-eclampsia has also been observed, which is why more research is needed on the subject to be able to resolve this dilemma.

Other micronutrients that have been linked to pre-eclampsia are vitamins A, E, C, and D, selenium, zinc, and folic acid, whose plasma concentrations are lower in women who suffer from it [11,197].

In summary, it seems clear that maternal nutritional status influences the natural development of pre-eclampsia. However, the interaction between nutritional factors and the development of this syndrome requires much more research.

#### 3.4.2. Gestational Diabetes Mellitus

Gestational diabetes mellitus is a common pregnancy complication that generally appears after week 20 and affects 5–10% of pregnant women. This pathology has short- and long-term health implications for both the mother and child [198]. 

Gestational diabetes mellitus refers to the subset of patients in which hyperglycemia is first diagnosed during the second or third trimester and excludes those women with overt diabetes that existed prior to pregnancy [199]. Its etiology is not well-understood, but pregnancy hormones are believed to decrease the body’s ability to use and respond to the action of insulin. During pregnancy, a state of hyperinsulinism occurs physiologically at the expense of human placental lactogen, a hormone similar to placental growth hormone. Estrogens, progesterone, and human placental lactogen stimulate insulin secretion, thereby increasing lipolysis and the concentration of free fatty acids in plasma, thus promoting resistance to the action of insulin. Maternal hyperglycemia stimulates the synthesis of insulin in the fetus, which is an important growth factor. For this reason, macrosomia is frequent in children of diabetic mothers. Furthermore, since hyperglycemia delays the fetal maturation process, these newborns run the risk of being weaker and presenting respiratory distress if they are born preterm [198].

In recent years, the relationship between gestational diabetes mellitus and diet has prompted investigators to examine nutritional supplementation as a potential prevention and treatment strategy for gestational diabetes mellitus. Nutritional supplements are an exciting avenue because they are typically well-tolerated, safe, and easy to administer—including during pregnancy [198].

A number of micronutrients have also been associated with gestational diabetes mellitus. 

A meta-analysis of seven intervention trials on gestational diabetes demonstrated that EPA and DHA reduced fasting blood sugar (RR 0.56, 95%CI 0.87 to 0.24) and HOMA-IR (RR 0.52, 95%CI −0.83 to −0.21) are statistically significant in comparison to placebo, while significance was narrowly missed in the cases of macrosomia (RR 0.48, 95% CI 0.22 to 1.02) and hyperbilirubinemia (RR 0.46, 95%CI 0.19 to 1.10) [200]. 

Decreased gestational plasma concentrations of vitamin C [201], vitamin D [202], vitamin E [203], vitamin B1 [204], vitamin B12 [205], selenium, and zinc [206] have been associated with gestational diabetes mellitus, although results across studies are conflicting. 

Vitamin D deficiency is arguably the micronutrient insufficiency most consistently associated with gestational diabetes mellitus. However, confounding variables, including ethnicity, sun exposure, and seasonality, can make it difficult to control for the independent relationship between vitamin D status and gestational diabetes mellitus, so the correlation remains uncertain [207].

#### 3.4.3. Nausea and Vomiting

Nausea and vomiting are common during the first months of pregnancy. Although vomiting usually disappears around week 16, nausea can continue until the end of pregnancy. Nausea and vomiting in pregnancy (NVP) may alter food intake, but the dietary and clinical consequences of NVP are poorly understood [208]. The causes of NVP are likely to involve hormonal, genetic, and gastrointestinal factors.

From a nutritional point of view, some strategies have been proposed for the prevention of nausea and vomiting in pregnancy [11].

- The periconceptional intake of multivitamin complexes has been shown to decrease the incidence of nausea and vomiting during pregnancy. Therefore, its administration is recommended for those women who have had nausea and vomiting in previous pregnancies [209].
- Based on the results from various studies, the American College of Obstetricians and Gynecologists (ACOG) recommends vitamin B6 intake under medical surveillance for nausea and vomiting during pregnancy [210].
- Eating frequent but not very abundant meals facilitates digestion and, in the case of vomiting, the losses of energy and nutrients are not so intense. In addition, foods of low volume and high nutrient density are recommended, as well as foods rich in carbohydrates that are better tolerated and easily digested.

Oral or intravenous thiamine (vitamin B1) supplements should be given to all women with prolonged vomiting [93,94]. However, it should be noted that, unfortunately, not all women have access to medical care. 

In Table 3, the different pathologies associated with pregnancy are summarized.

## 4. Conclusions

As has been discussed throughout this review, there is evidence that micronutrient deficiencies negatively affect maternal health and the outcome of pregnancy. Importantly, no single micronutrient is responsible for adverse effects. For this reason, supplementing to correct one deficiency will not be very effective while other deficiencies exist.

The results of controlled supplementation studies for all relevant micronutrients together have not been published to date. For now, it seems impossible to predict the possible effect of a multivitamin and mineral supplement that meets all the needs of pregnant women. However, there are some preparations on the market that include adequate doses of folic acid, vitamin B12, iron, iodine, and small amounts of other micronutrients that meet the needs of most pregnant women.

It should be taken into account that most of the effects and results of supplementation that have been described refer to subjects with serious deficiencies, making it difficult to generalize to the general population. Furthermore, most of the controlled studies on supplementation have been conducted in industrialized countries, where deficiencies are less frequent. Thus, the observed effects could represent an underestimation of what is expected in developing countries.

Furthermore, the causes or at least the potential mechanisms that explain the relationship between micronutrient intake during the periconceptional period and the development of pregnancy should be well-defined and understood. From all this, it is deduced that there is a need to carry out more experimental and interventional studies on humans in order to adjust the recommended daily values and, accordingly, the recommended periconceptional nutrition for the mother.

It is important to promote supplementation through health education and launch health campaigns aimed at women of reproductive age.

### General Recommendations

- Proper nutrition of women should be considered as a goal to prevent nutritional imbalances that interfere with pregnancy outcomes. In particular, diet during the first trimester may be more important for the development and differentiation of various organs. In addition, good nutrition prior to conception is essential for an optimal start and development of pregnancy. Since the nutritional intake of women of childbearing age during the preconception period seems to be inadequate mainly with regard to micronutrients, efforts should be increased to promote a healthy diet and lifestyle, not only during pregnancy but also before, since pregnancies are often unplanned.
- Supplementation with 770 retinol equivalents [11] is recommended in women with deficits or at risk of pre-eclampsia [36].
- Supplementation with folic acid is the most important and effective intervention for the reduction of congenital neural tube defects. It is recommended to take a supplement with 0.4 mg a day in the month prior to conception, or at least during the first trimester. In the case of a previous history of malformations, the dose will be increased to 4 mg per day.
- In general, low-dose oral iron supplementation is recommended during the second half of pregnancy in women without risk of iron deficiency. In patients with previous anemia, it should be started early in pregnancy.
- Depending on women’s current iodine levels, iodine intake should be increased using iodized salt and a supplement of 200 µg per day, starting before conception, in the same way as with folates. It should be maintained throughout pregnancy and lactation. This recommendation is endorsed by most scientific societies.
- Calcium is not routinely recommended except in high-risk women: pregnant women from developing countries, under 18 years of age, with high risk of pre-eclampsia, or with poor intake. The diet should include at least three servings of foods rich in calcium.
- A daily intake of 200 mg of ω3 polyunsaturated fatty acids should be sought.
- The correct intake of vitamin D should be ensured in those patients at risk of developing deficiencies (low sun exposure) through diet (milk, fortified dairy products, and fatty fish) or through nutritional supplements if necessary [11,47]. In addition, there are studies that recommend its supplementation for the prevention of gestational diabetes, neonatal hypocalcemia, and low birth weight.
- The American Cancer Society proposes vitamin K supplementation in pregnant women taking anticonvulsant medications or in those who have suffered from cholestasis.
- Supplementation with vitamin E in older women and with vitamin C in smokers could be considered.

## Figures and Tables

**Table 1:** Search process.

	Principal Term		Synonyms		Synonyms	
Pregnant women	Pregnancy [Mesh]	OR	Maternal–fetal health [Mesh]	OR		AND
Micronutrients	Supplementation	OR	Minerals [Mesh]	OR	Vitamins [Mesh]	AND
Any intervention						AND
Recommendations	Requirement	OR	Deficiency of nutrients	OR		AND

**Table 2:** Recommended daily dose of vitamins in women.

Daily Doses of Nutrients Recommended for Women		
Vitamin	Not Pregnant	Pregnant
B1 (Thiamine) (mg) [11,90,91,92,93]	1.1	1.4
B2 (Riboflavin) (mg) [11,42]	1.1	1.4
B3 (Niacin) (mg) [42,88]	14	18
B6 (Pyrodaxine) (mg) [11,83,84,85,86,87]	1.3	2
Folic acid or B9 (µg) [98,99]	200	400
B12 (µg) [100,101,102,103,109,110,111]	2.4	2.6
Vitamin A (µg RE **) [24,30,31,32,33,34,35]	700	770
Vitamin C (mg) [11,40,41]	75	85
Vitamin D (µg) [47,54,55]	2	5
Vitamin E (mg) [44,45]	15 (USA) 10 (Europe)	15 (USA) 10 (Europe)
Vitamin K (µg) [13,78,80]	60–65	65

** RE = retinol equivalents.

**Table 3:** Pathologies associated with nutritional deficits during pregnancy.

Pathology	Associated Deficit
Pre-eclampsia	Vitamin A [36] and calcium [196] Vitamins C [11], E [36], D [58,60,61,62,63,64], and B6 [42] Folic acid and B12 [104,105] Magnesium [125,197] and zinc [36] Fatty acids ω3 [182,183,184,185,186]
Low weight at birth	Vitamin D [47] Vitamins A [24,25], E [43], and B6 [42,84,85,86,87] Magnesium, zinc [36,151,152], and iron [139,140]
Congenital malformations	Folic acid [9,11,95,96,97] Vitamins B6 [82,83,84,85] and B12 [101,102,103,104,105,114,115,116]
Gestational diabetes mellitus	Vitamin D [202,207]
Nausea and vomiting	Vitamin B6 [93,94]
Prematurity	Calcium [9,122,123]
